# Investigating the effect of electrical brain stimulation using a connectome-based brain network model

**DOI:** 10.1186/1471-2202-16-S1-O13

**Published:** 2015-12-18

**Authors:** Tim Kunze, Alexander Hunold, Jens Haueisen, Viktor Jirsa, Andreas Spiegler

**Affiliations:** 1 Institute of Biomedical Engineering and Informatics, Ilmenau University of Technology, Ilmenau, 98693, Germany; 2 Max Planck Institute for Human Cognitive and Brain Sciences, Leipzig, Germany; 3 Aix-Marseille Université, Institut de Neurosciences des Systèmes, Marseille, France; 4 Institut National de la Santé et de la Recherche Médical, UMR_S 1106, 27 Bd Jean Moulin, 13385, Marseille Cedex 5, France; 5 Centre National de la Recherche Scientifique, 3, rue Michel-Ange, 75794, Paris, France

## 

Transcranial direct current stimulation (tDCS) leads to positive effects in neurological and psychiatric diseases, such as depression, pain, or stroke, which outlast the treatment itself. Although numerous influencing stimulation parameters and factors are known, the mechanisms behind tDCS remain unclear. To reveal the mechanisms tDCS started to be considered to affect networks while (de)polarizing parts of the brain. We study here the ability of tDCS as a tool to bias functional networks by affecting the connections given the brain structure.

We used structural data, that is, a human connectome to construct a large-scale brain network model of 74 cerebral areas, each described by a Jansen and Rit model. The model was designed on the basis of the neuroinformatics platform The Virtual Brain to account for reproducibility of the simulations. The tDCS-induced currents on the cerebral areas were calculated using a finite element method model. Based on the dynamical repertoire of an isolated area [1], we analyzed the brain activity, that is, the spatiotemporal dynamics in terms of rhythms and baseline potentials during rest, during tDCS, and the change between both.

**Figure 1:**
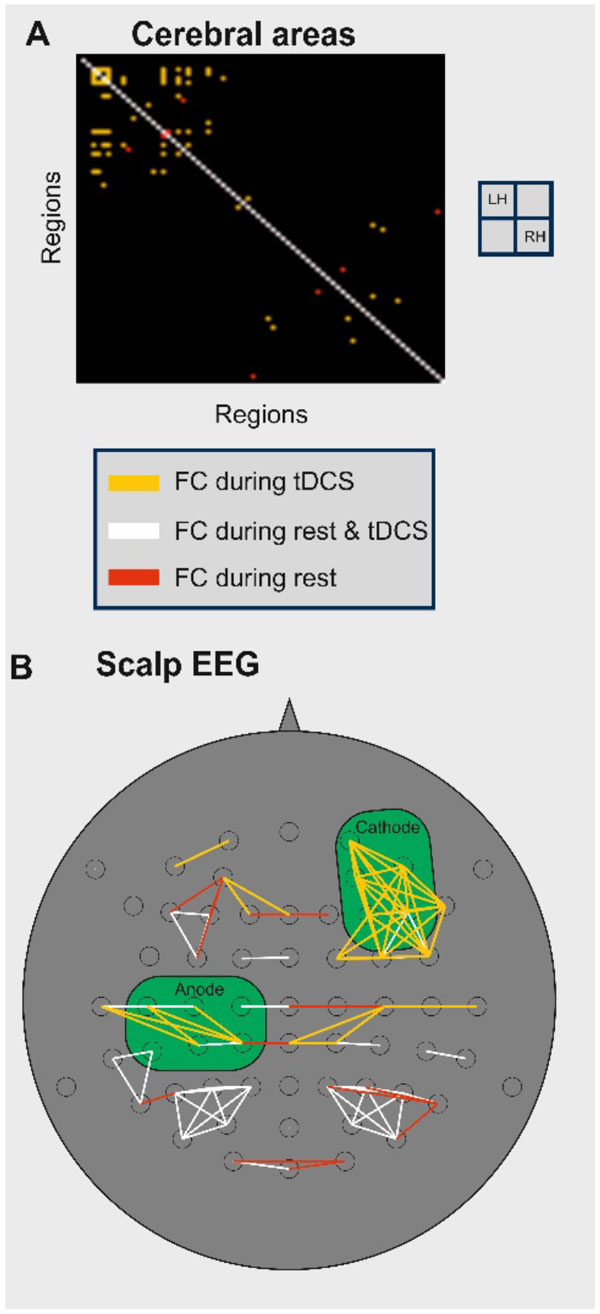
**New functional connections are established during tDCS: among cortical areas, Panel A; and among scalp EEG electrodes, Panel B**.

We identified the network states during rest and catalogued all states for further modeling studies. During tDCS, increased functional connectivity was found among a set of scalp EEG sensors, as reported in measurements [2], as well as among cerebral cortical areas (see Figure 1). Furthermore, tDCS led to sharpened frequency spectra and increased (anode) or decreased (cathode) power in the respective areas.

This study supports the notion that noninvasive brain stimulation is able to bias brain dynamics by affecting the competitive interplay of functional subnetworks. Our work constitutes a basis for further modeling studies to test target-oriented manipulation of functional networks (e.g. through adapted electrode montages) to improve pertinent treatment conditions. Furthermore, our approach emphasizes the role of structural data such as the network topology in emerging dynamics. Dynamics cannot necessarily be predicted from the structure but we found the structure especially important at transitions of network states.
